# Is Contraception Injurious to Health?

**Published:** 1916-02

**Authors:** 


### Is Contraception Injurious to Health?

Although this question has been answered pretty thoroughly in "The Small Family System," it is necessary to refer to it from time to time on account of its great importance, and because of the new attacks and new evidence which the lapse of time bring forth. Recently Dr. Dulberg, in the Manchester *Umpire,* has repeated the conventional declaration concerning the physiological and moral evils of prevention. We also have before us a volume, entitled "The Conjugal Relationships as to Health," by Dr. A. K. Gardner, Professor of Clinical Midwifery in New York Medical College, now reaching its twentieth thousand, and praised by doctors, clergymen, and the press in the United States, which contains the best attempt at making out a case against preventive devices we have yet seen. It comes rather opportunely on the heels of Dr. W. J. Robinson's challege in his "Limitation of Offspring" (reviewed in our October issue):

"I challenge any physician, any gynecologist, to bring forth *a*

*single* authenticated case in which disease or injury resulted from the use of the modern methods of prevention. I know they cannot do it."

Dr. Gardner's remarks are the more worthy of consideration, as he appears fully to appreciate the necessity for the avoidance of procreation on economic and eugenic grounds in many cases. Indeed, to those of his medical brethren who prescribe early marriage for physiological reasons he contends that (page 57):

"An unseasonable or premature marriage, relatively to the social economy, is a cause of disorder, misery, and despair, which increases without cessation, and multiplies as the new family increases."

On the other hand, Dr. Gardner is by no means free from conventional and theological prejudice, which considerably discounts the value of his arguments. He takes the view that continence is necessary and uninjurious, and supports this view by medical opinion entirely on one side. He does not even seem to be aware of the early experiences of the monasteries, or of the modern sexological investigators. Our point, as neo-Malthusians, is that whether sex-abstinence is possible for mature adults or not, it has almost universally broken down in practice, with terrible consequences; and it is so severe a deprivation that no one should be called upon to endure it if there are any healthful means of avoiding it without bringing children into the world for whom there is no satisfactory prospect of health and happiness. Whatever restraint and sacrifice may be really necessary for the welfare of humanity, we should all do our best to make as cheerfully as possible; but there are far too many opportunities of this kind in life for us to uphold a single unnecessary one.

Dr. Gardner quotes the following figures from Mayer as to the mortality among the religious orders of both sexes who have taken vows of chastity (page 41):

| | Religious Orders. | Laity. |
|---|---|---|
| In the ten years between 16 and 25 | 2.68% | 1.48% |
| In the ten years between 31 and 40 | 4.40% | 2.74% |

Attempts are made in the book to show that these figures do not prove the injurious effects of continence, including the naïve confession that we dare not guarantee that the rules of chastity are not infringed upon in the religious orders. When we consider the sheltered lives of these devotees, however, it is difficult to avoid the conclusion that the deprivation of natural sex gratification is seriously detrimental to their health. Dr. Gardner himself appreciates this when he says (page 59):

"It is only impossible not to admit, as everything indicates, that marriage, resulting in the satisfaction of venereal desires, exerts a favorable influence upon the health of women, and serves to prolong their lives. How otherwise can we explain the notable difference of mortality which exists between married and unmarried women during the time when they ordinarily become mothers, say between 20 and 45, a difference which, from late statistics, amounts to 29 per cent in favor of marriage?"

How, indeed? Notice that these are "late statistics," presumably American ones, so that they indicate that among American married women, who now nearly all have small families, and even appear to have extensive recourse to the admitted dangers of abortion, in ignorance of satisfactory preventive methods, they have the advantage by 29 per cent over their unmarried sisters. Surely this in itself is amply sufficient to prove that sex union, even with the dangers of unintelligent interference, and of the venereal dissease which is so common in America, is a most highly advantageous thing for women.

Although Dr. Gardner appears genuinely anxious to be impartial, and his book is an immense improvement upon the generality of such productions (notably upon the wholesale diatribes of the late Dr. F. W. Taylor), his theological bias is unmistakable, so that when we come to Chapter VII, entitled "Methods Used to Prevent Conception, and Their Consequences," we are not supprised to find the following:

"The means used for preventing conception, more particularly affecting women, together with the more bodily injurious, more nervously exhausting, and more sinfully demoralizing procedures for the purpose of destroying and making away with the results of conception, these are the crying evils of the age and of the world."

It is satisfactory to find that Dr. Gardner is in advance of the generality of traducers of contraception in separating it from abortion, at least in degree; but we wonder how far he realizes that the latter has been the "crying evil" of every age, and that it certainly has not been the result of contraceptive propaganda? The bulk of his arguments are of an *a priori*, reasoning-from-analogy, order, and he again betrays his bias in his reference to the biblical story of Onan. But in this chapter he has done what we do not believe any opponent of contraception has done before—he has given us records of three of his actual cases which appear to sustain his contention that married couples should either let matters take their normal course or abstain from intercourse if they do not desire children. In these cases he cites the experience of three husbands who had lost their health, presumably as the result of contraceptive measures, and who were completely restored by abstinence, or by confining relations to a supposedly safe period. One of these cases looks like one of previous excess, and in another the prevention was by a method which the majority of medical advocates of prevention admit to be injurious to certain organizations. In the third case we are not informed as to the method, nor are we told in any of the cases that the alternative prescription of abstinence was actually followed.

This is all the evidence we have as regards men. Concerning women the author tells us (page 97):

"We have at our disposition numerous facts which rigorously prove the disastrous effect of abnormal [intercourse] to the woman, but we think it useless to publish them. All practitioners have more or less observed them, and it will only be necessary for them to call upon their memories to supply what our silence leaves."

In view of Dr. Robinson's challenge, and of the extreme importance of the subject, may we urge upon Dr. Gardner that he *should* give us such facts? Nothing but facts and a rigorous proof derived from them will weigh against the overwhelming mass of general evidence in favor of contraception. We neo-Malthusians are only anxious for the truth, and we shall be the first to acknowledge the validity of any real evidence on the other side; but Dr. Gardner must agree that what he has given us is indeed slender proof of such an all-important assertion.

In view also of the undoubted "disastrous effects" of too numerous and too rapidly recurring pregnancies upon women, of which "all practitioners" have abundant evidence, and of which our letters from struggling parents bear such poignant witness, we must be excused from holding somewhat strong opinions of those medical men who attempt to terrify them by vague generalities.

Here is a brief *résumé* of the evidence for the hygienic justification of contraception:

A. Facts.

1. The great fall in the birth rate which has taken place in very many countries, and which is admitted on all hands to be chiefly due to the adoption of contraceptive measures, has been accompanied by a great drop in the death rate and a remarkable increase of the mean duration of life, which has not occurred in countries where the birth-rate has not fallen. This is almost conclusive evidence that great benefit to the general health of the community has resulted from the adoption of contraception, though we are quite willing to agree that it is mainly due to the relief of economic pressure. Nothing, however, can do away with the fact that there has been an enormous improvement in longevity, which should not have occurred if contraceptive measures were in any way seriously injurious.

2. The death rate of both men and women at the reproductive ages has most markedly diminished, and is much lower than among the unmarried, as Dr. Gardner admits.

3. Before the spread of contraceptive knowledge through the Knowlton trial of 1876, the families of medical men in England averaged close on five children. Now they rarely exceed two or three. Enquiries among the medical men of Paris have shown that they extensively practice "conjugal Onanism," and are quite unconscious of evil effects.

4. The experience of the various neo-Malthusian Leagues, of numerous cases in which contraceptive measures have been practiced over long periods without the slightest appearance of bad effects.

5. The notable success of the Dutch neo-Malthusian movement, the only one which has been able to popularize the employment of the most approved preventive methods. The death rates of The Hague and Amsterdam, the chief centers of this propaganda, are now the lowest of any cities in Europe.

6. The remarkably low death rate of New Zealand, where, according to statements made by the Chairman of a Clerical Conference, preventives have been freely hawked from door to door.

B. The opinions of eminent medical authorities in various countries, including the eminent gynecologist, Dr. Hector Treub; the ex-President of the American Medical Association, Dr. A. Jacobi; the well known sexologists, Dr. Havelock Ellis, Professor Forel, Dr. Iwan Bloch, Drs. Max and Julian Marcuse, Dr. Anton Nyström, and last, but not least, Dr. W. J. Robinson. Also the report of the Hungarian Medical Senate and the report of Dr. Bond to the Leicester Ruri-Decanal Conference.

Again, we have the striking fact that, despite the enormous spread of family limitation, and the obviously hostile attitude of the medical profession to it, there has never been, we believe, a single record of cases brought before a medical meeting or congress to show the harmful effects of contraceptive devices. Ten years or more ago we had thunders of denunciation, but no proofs. Year by year, as the birth rate has fallen, the denunciations have weakened, and at the last great International Medical Congress in London not a single reference was made to the subject beyond the paper by Dr. Hall on the use of lead plaster to produce abortion. In an age of new diseases, when cases are brought forward of nervous disorders from motor bicycling, tube traveling, etc., not a solitary physician has come forward with cases of disease attributed to contraception, although millions of persons are practicing it. Meanwhile the birth rate goes on falling—and the death rate with it. If the opponents of contraception have really any evidence to give us, let them now speak, or for ever after hold their peace.—*C. V. Drysdale, B. Sc., in the Malthusian, December 15th.*

---

Clinics will be held at Dallas hospitals on the third Thursday of each month, beginning January 20th, by physicians and surgeons of the Dallas County Medical Society, according to a new plan agreed upon at a meeting at the Baylor Medical College.

---

### New Methods of Surgery.

Recent advances in surgery were illustrated in connection with the sessions of the Clinic Congress of Surgery of North America, which was held in Boston the last of October. A new method of trephining the skull, a perfected operation for cataract and an improved system of blood transfusion were features of the clinics.

Dr. Harvey Cushing, of Boston, performed the trephining operation, which, it is claimed, obviates the necessity of inserting metal plates in the skull.

In transfusing the blood of a healthy person to the veins of a patient, it is no longer necessary for the two to be together, another operation demonstrated today. The blood of the healthy person was placed in a wax-lined tube, and at the surgeon's convenience conducted to the veins of the patient. This method, it was pointed out, means facility of operating, no destruction to the radial artery of the healthy person, no danger of blood clotting while operating, and the possibility of exactly measuring the amount of blood to be transfused.

Improvements in operating upon cataract, it was said, reduce the time of the patient's blindness, because it is now no longer necessary for the victim to wait for the cataract to "open," a process that sometimes required several years.

